# Effects of Hydrodynamic Ozonated Water Processing on the Thermal Stability and Structural Integrity of the Human Amniotic Membrane

**DOI:** 10.3390/jfb17070352

**Published:** 2026-07-20

**Authors:** Marcia Guelma Santos Belfort, Francisco Dimitre Rodrigo Pereira Santos, Maycon Crispim de Oliveira Carvalho, Aline Casarin dos Santos, Pedro Augusto Laurindo Igreja Marrafa, João Gomes de Oliveira Neto, Carlos José de Lima, Adriana Barrinha Fernandes

**Affiliations:** 1 Postgraduate Program in Biomedical Engineering, Biomedical Engineering Institute, Universidade Anhembi Morumbi (UAM), São José dos Campos 12230-002, SP, Brazil; 125111209186@ulife.com.br (M.C.d.O.C.); 125221101542@ulife.com.br (P.A.L.I.M.); adriana.morett@ulife.com.br (A.B.F.); 2 Instituto de Ensino Superior do Sul do Maranhão (IESMA), Unidade de Ensino Superior do Sul do Maranhão (Unisulma), Imperatriz 65907-070, MA, Brazil; 3 Instituto de Estudos em Saúde e Biológicas (IESB), Universidade Federal do Sul e Sudeste do Pará (Unifesspa), Marabá 68507-590, PA, Brazil; francisco.dimitre@unifesspa.edu.br; 4 Centro de Inovação, Tecnologia e Educação (CITÉ), São José dos Campos 12230-002, SP, Brazil; 5 Postgraduate Program in Materials Science, Universidade Federal do Maranhão (UFMA), Imperatriz 65915-060, MA, Brazil; joao.gon@ufma.br

**Keywords:** human amniotic membrane, FT-IR, differential scanning calorimetry, thermogravimetry

## Abstract

This study aimed to verify the morphology, biochemical composition, and thermal characterization of hydrated human amniotic membrane (HAM) processed in an ozonated water hydrodynamic system. This is an in vitro experimental study in which HAM samples were divided into two groups: in natura (IN) and ozonated (O_3_). Analyses were performed using histology, Fourier-transform infrared spectroscopy (FT-IR), thermogravimetric analysis (TGA/DTG), and differential scanning calorimetry (DSC/dDSC). Ozonation for 40 min preserved the biochemical integrity of HAM, maintaining the characteristic vibrational bands of Amides I, II, and III. Histological analysis showed morphological changes in epithelial cells, with partial removal in some regions, while the basement membrane and the scaffold remained preserved. Thermal analysis revealed that the in natura sample presented a bimodal dehydration profile, with a first event occurring between 60 and 65 °C associated with the evaporation of free or weakly bound water, and a second event peaking around 80 °C related to the removal of structural water. In contrast, the ozonated HAM exhibited a unimodal profile, with the mass loss peak shifted to approximately 70 °C. These findings were corroborated by DSC analysis, which showed a reduction in denaturation temperature from approximately 85 °C in the in natura sample to around 75 °C in the ozonated sample. The dDSC analysis confirmed the transition from a bimodal to a unimodal behavior after treatment, indicating a reduced energy barrier for protein denaturation and lower thermal stability of the collagen matrix. These results suggest that ozonation promotes alterations in the epithelial layer, which may favor the loss of both free and bound water. It is concluded that processing with ozonated water induces structural modifications, especially in the epithelial layer, and reduces the thermal stability of hydrated HAM without significantly altering the biochemical signature of collagen. This approach shows potential as an alternative method for membrane processing; however, functional evaluations are required to confirm its clinical applicability.

## 1. Introduction

The human amniotic membrane (HAM) is one of the fetal structures located on the inner part of the placenta, which is usually discarded after childbirth [1]. Structurally, its thickness ranges from 0.02 to 0.5 mm and it is composed of three main histological layers: the epithelial layer, the basement membrane, and the stroma [2]. The surface of the HAM consists of a single layer of cuboidal epithelial cells firmly adhered to a basement membrane composed primarily of types IV and VII collagen, laminin, and fibronectin [3,4,5]. In turn, the stroma comprises three layers organized into: a compact layer composed of types I, III, and IV collagen and fibronectin; a fibroblastic layer rich in fibroblasts, types I, III, and VI collagen, laminin, and fibronectin; and a spongy layer consisting of type III collagen and proteoglycans. Due to this morphological organization and biochemical composition, HAM has been extensively studied as a potential biomaterial [3].

The HAM has been increasingly used in the production of biological dressings [6,7,8] and in clinical practice due to its regenerative and healing properties, which confer characteristics of great interest for regenerative medicine [2]. Its properties include anti-inflammatory, antibacterial, and immunological effects, as well as the ability to promote re-epithelialization, thereby reducing the scarring and inflammatory processes, in addition to exhibiting antiangiogenic characteristics [9].

Since it is a biological tissue, the sterilization process is fundamental prior to its clinical use, given that microbial contamination is one of the main reasons for HAM disposal, with bacteria being the most frequent agents [10]. Gamma radiation is currently considered the gold-standard method for tissue sterilization, most often employed at doses of 25 kGy. This value was established because it represents approximately 40% above the minimum dose required to inactivate the most resistant microorganisms, ensuring greater microbiological safety [11].

However, irradiation at doses ≥ 20 kGy can lead to morphological and structural alterations in the HAM, particularly regarding the disintegration of the basement membrane, decomposition of thin collagen fibers, and condensation of nuclear chromatin. Irradiation can also lead to a significant decrease in growth factors, such as Epithelial Growth Factor (EGF) or Fibroblast Growth Factor (β-FGF), as well as a significant reduction in Tissue Inhibitors of Metalloproteinase 1, 2, and 4 (TIMPs) [12].

The various limitations of current sterilization methods, especially for biomaterials, have stimulated the development of alternative techniques. The continuous search for low-temperature sterilization technologies stems from the need to adapt sterilizing agents to the physicochemical and biological characteristics of biomaterials, the convenience of faster processing, and environmental appeals compared to conventionally used methods [13].

Recent studies have demonstrated the feasibility of a new method for HAM disinfection and sterilization based on an ozonated water hydrodynamic system. Ozone (O_3_) is a triatomic oxygen molecule that generates oxidizing compounds upon decomposition. When dissolved in water under controlled flow, it has proven effective in microbial inactivation while preserving essential structural characteristics of the treated tissue. The main advantages of using ozonated water include low toxicity to the operator and the fact that it does not generate environmental residues, allowing it to be discharged into the sewage system without the need for additional treatment. Notably, to date, no microbial resistance to O_3_ has been described in the literature [14,15].

The physicochemical characterization of collagenous biomaterials has been routinely performed using thermal analysis techniques, which provide important information regarding structural stability, water interaction, and thermal degradation behavior. Among these techniques, Thermogravimetric Analysis (TGA) and Differential Scanning Calorimetry (DSC) stand out, being widely used in the investigation of collagen-rich tissues and extracellular matrix-derived biomaterials [16]. TGA allows for the evaluation of sample mass variation as a function of temperature, making it possible to identify processes such as dehydration, the release of structural water, and the thermal degradation stages of the organic matrix [17]. On the other hand, DSC provides data on thermal transitions associated with the stability of the collagen molecular structure, including endothermic events related to the denaturation of the triple helix and the disruption of intermolecular interactions stabilized by hydrogen bonds [18]. Therefore, the combination of TGA and DSC constitutes an important approach for the characterization of collagen-based biomaterials, such as HAM, contributing to the understanding of their physicochemical properties and the evaluation of their suitability for biomedical applications.

Based on the previous study by Botelho et al. [15], in which 40 min of exposure to a hydrodynamic ozonized water system was demonstrated to be sufficient to achieve effective sterilization of HAM, the present study was designed as a complementary investigation focused on evaluating the potential effects of this process on the physicochemical and structural properties of the biomaterial. Therefore, this study aimed to verify the morphology, biochemical composition, and thermal characterization of hydrated HAM processed in an ozonated water hydrodynamic system using histological analysis, Fourier-transform infrared spectroscopy (FT-IR), thermogravimetric analysis (TGA), and differential scanning calorimetry (DSC), in order to investigate possible changes in extracellular matrix organization, thermal stability, and biomaterial morphology.

## 2. Materials and Methods

### 2.1. Study Design and Ethical-Legal Aspects

This research consisted of an in vitro experimental study approved by the Human Research Ethics Committee of Universidade Anhembi Morumbi under opinion number 3.984.423, CAAE: 289370020.0.0000.5492. The study was conducted in accordance with the guidelines established by Resolution of the Collegiate Board N. 190, dated 18 July 2003, of the Agência Nacional de Vigilância Sanitária (ANVISA), which defines the technical standards for the operation of umbilical cord and placental blood banks [19].

### 2.2. Sample Collection

The HAM collection was performed in the surgical center of Santa Casa de Misericórdia de Pindamonhangaba, and the samples were obtained from donors who voluntarily signed the Informed Consent Form (ICF).

Fresh HAM samples were aseptically obtained from cesarean deliveries of seronegative donors (for syphilis, hepatitis B, toxoplasmosis, and HIV), with a mean age of 30 years (range: 28–32 years). During the selection process, membranes presenting any visible structural damage were excluded from the study, ensuring the visual integrity of the analyzed material.

The HAM was immediately placed in a glass container containing sterile saline solution (0.9% NaCl) and subsequently stored in a thermal container at a controlled temperature ranging from 10 to 15 °C. Thereafter, the samples were transported to the Centro de Inovação, Tecnologia e Educação, where they underwent a manual washing process using sterile solution in ten consecutive repetitions to remove any persistent biological residues.

Two HAM obtained from independent donors were included in this study. Following collection and processing, each membrane was subdivided into fragments and allocated into two groups: one designated as in natura (IN) and the other as ozonated (O_3_).

### 2.3. Experimental Protocol

**Figure 1:**
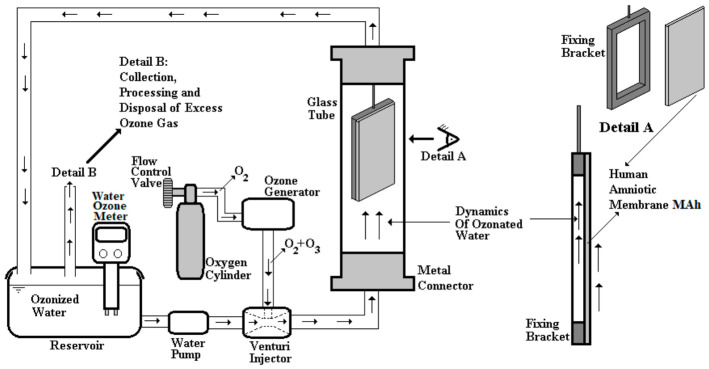
Schematic apparatus for HAM ozonation using a hydraulic circuit with ozonated water via a Venturi injector. Prior to sample insertion, the water was ozonated for 15 min, during which the ozone concentration in the water was measured using a TIZ-OEM meter (Anseros, Tübingen, Germany). The entire system was installed inside a fume hood with gas exhaust, where excess ozone gas was collected directly at the exhaust inlet. At this location, hot air adjusted to 50 °C was also introduced to rapidly degrade the ozone before its release into the external environment, as shown in Detail B.

The experimental protocol adopted in this study was based on the studies by Awoyama et al. [14] and Botelho et al. [15], in which a closed hydrodynamic system producing ozonated water was used. Prior to the procedure involving the amniotic membrane, 1 L of water was ozonated using a closed hydraulic circuit with a centrifugal pump coupled to a Venturi injector to incorporate ozone gas (Figure 1). The system also included a head assembly composed of a glass tube and two cylindrical stainless-steel components designed for the subsequent placement of the HAM, as shown in Detail A in Figure 1.

Initially, the water was ozonated for 15 min, during which the ozone concentration in the water was measured. Subsequently, the HAM sample was inserted into the system and subjected to ozonation for 40 min.

The ozone generator used (MS3G, MS Ltd.a., Rio de Janeiro, Brazil) was adjusted to a concentration of 58 mg/L, while the oxygen gas flow regulating valve was set to 0.25 L/min. Consequently, the Ozone Feed Rate reached 11.3 mg/min. Considering that the sample area measured 52 cm^2^ and considering the dynamic action of the ozonated water over the HAM surfaces, the Applied Ozone Dosage corresponded to 8.7 mg(O_3_)/cm^2^ [20].

The concentration of dissolved ozone was experimentally measured at predefined time intervals during the ozonation process. Several regression models were evaluated to describe the concentration profile, including exponential and polynomial functions. Among the tested models, the fifth-order polynomial regression provided the best fit to the experimental data (R^2^ = 0.992) and was therefore selected as an empirical mathematical model for interpolation purposes. The resulting equation was used to estimate ozone concentration at intermediate time points within the experimental range and does not imply a mechanistic description of the ozone dissolution process.

### 2.4. Analyses

#### 2.4.1. Fourier Transform Infrared Spectroscopy (FT-IR) Analysis

FT-IR spectra were obtained using a Spectrometer Frontier FT-IR/NIR (PerkinElmer, Shelton, CT, USA), operating within the spectral range of 400–4000 cm^−1^, with 45 scans and a spectral resolution of 4 cm^−1^. The system was equipped with a Universal Attenuated Total Reflection (UATR) accessory. Spectra were acquired in quintuplicate, and subsequently, the median of the spectra was determined for quantitative and qualitative analyses.

The spectra were processed and plotted using OriginPro software version 8.5 (OriginLab Corporation, Northampton, MA, USA), allowing systematic comparison of the spectral profiles among the different experimental groups.

#### 2.4.2. Histological Analysis

Tissue samples were fixed in 10% formalin solution to preserve tissue structure, paraffin-embedded, and sectioned at a thickness of 5 µm using a microtome. For each sample, two histological slides were prepared, each containing approximately eight sections After obtaining the histological sections, the slides were stained with hematoxylin and eosin, and images were acquired using T Capture^®^ software, version 4.9.

#### 2.4.3. Thermoanalytical Analyses: Thermogravimetric Analysis (TGA/DTG) and Differential Scanning Calorimetry (DSC/dDSC)

The TGA and DSC curves were obtained using the Q-600 thermogravimetric module (TA Instruments, New Castle, DE, USA), which enables simultaneous measurement of mass variation and heat flow within the same sample. The experiments were conducted using alumina and aluminum crucibles under a nitrogen gas flow of 50 mL/min, ensuring a controlled atmosphere.

The heating rate was set at 10 °C/min, with a final temperature of 150 °C, followed by a 10 min isothermal stage for thermal stabilization of the sample. The samples were placed in partially sealed aluminum capsules containing approximately 15 mg of material, with a weighing precision of ±1 mg. Thermal analysis was performed within the temperature range of 0–150 °C, allowing detailed evaluation of membrane behavior under controlled heating conditions.

The analysis of thermal events was performed using DTG and dDSC (first derivative) curves processed in OriginPro 2018 software version 8.5 (OriginLab Corporation, Northampton, MA, USA). To improve the definition of degradation peaks, an adjacent averaging smoothing filter (20 points) was applied, ensuring preservation of the thermal profile integrity and accuracy in determining the temperatures corresponding to the maximum mass loss rate (Tmax).

Due to the descriptive nature of the thermal characterization (TGA and DSC), data were analyzed comparatively based on the identification of reproducible thermal events, including mass loss stages and denaturation peaks. Analyses were performed in duplicate to verify reproducibility of the thermal profiles. No inferential statistical analysis was applied, as the primary purpose of these techniques was physicochemical characterization rather than hypothesis testing.

## 3. Results

### 3.1. Dissolved O_3_ Concentration Curve in Water

**Figure 2:**
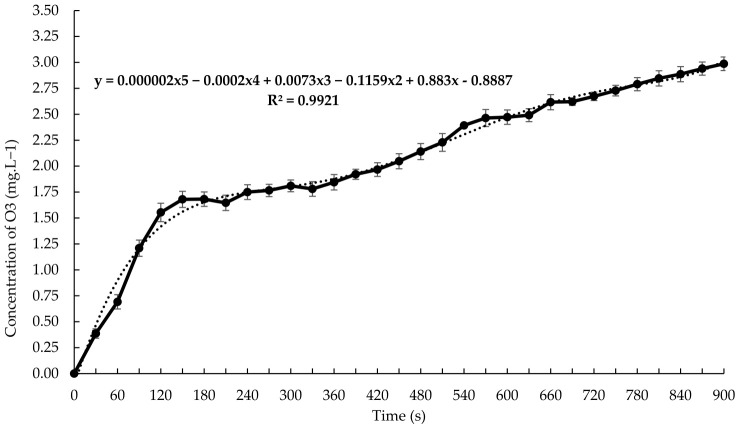
Curve of dissolved O_3_ concentration in water at 20 °C versus time (s).

Figure 2 illustrates the dissolved O_3_ concentration curve in water over 900 s (15 min) of ozonation at 20 °C. It was observed that during the first 180 s (3 min), the O_3_ concentration increased rapidly, characterizing the initial phase of intense gas diffusion into the liquid medium. After this period, the increase in concentration became slower, reaching a concentration of 2.99 mg/L at 900 s (15 min). Different regression models were evaluated, and the fifth-order polynomial provided the best fit to the experimental data, presenting the highest coefficient of determination (R^2^ = 0.992). The fitted curve was used exclusively for interpolation of experimental data and does not represent a mechanistic model of ozone dissolution kinetics.

The dissolved O_3_ concentration in water reached 2.67 mg/L after 720 s, indicating that the target ozone concentration had already been achieved. However, the ozonation process was maintained for a total of 15 min before insertion of HAM, according to the standardized treatment protocol described by Botelho et al. [15].

### 3.2. FT-IR Analysis

**Figure 3:**
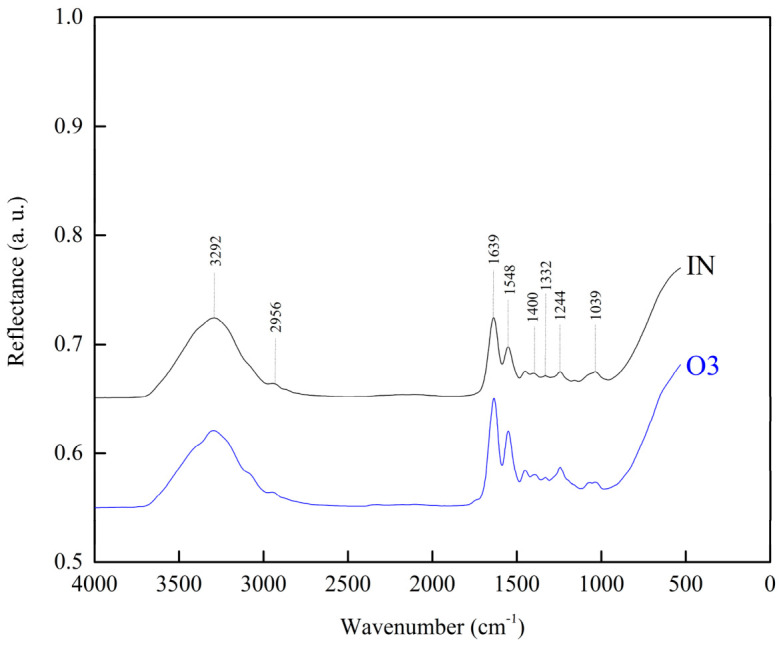
FT-IR-UATR spectra of in natura (IN) and ozonated (O_3_) Human Amniotic Membrane (HAM). The main absorption bands were assigned to O–H/N–H stretching (~3292 cm^−1^, Amide A), asymmetric C–H stretching (~2956 cm^−1^), Amide I (~1639 cm^−1^, mainly C=O stretching of peptide bonds), Amide II (~1548 cm^−1^, N–H bending and C–N stretching), CH_2_ bending (~1400 cm^−1^), Amide III (~1244 cm^−1^, collagen-associated vibrations), and C–O/PO_2_^−^ stretching (~1039 cm^−1^), associated with carbohydrates and nucleic acids.

Figure 3 presents the FT-IR spectra obtained for the IN and O_3_ samples. The spectra allowed the identification of characteristic bands associated with the main biomolecules present in the membrane, including lipids, carbohydrates, proteins, and nucleic acids (Table 1).

The qualitative spectral characteristics of the IN and O_3_ samples were similar (Figure 3). Eight characteristic absorption bands were identified at frequencies of 3292, 2956, 1639, 1548, 1454, 1400, 1244, and 1039 cm^−1^. Subtle variations were observed, mainly expressed by changes in peak intensity. The O_3_ treated samples exhibited greater intensity at the peaks of 1630, 1548, and 1244 cm^−1^, which are related to Amide I, Amide II, and Amide III, respectively, compared with the IN samples.

The absorption band between 1600 and 1640 cm^−1^ corresponds to protein Amide I, mainly attributed to C=O stretching [21,22]. In contrast, the absorption band between 1510 and 1560 cm^−1^ corresponds to the Amide II band, attributed to N–H bending and C–N stretching modes [21,22,23]. Peaks around 1210–1300 cm^−1^ are assigned to Amide III, which results from the in-phase combination of C–N stretching and in-plane N–H bending, with some contribution from C–C stretching and C=O bending vibrations [21,24]. The spectral band at 1454 cm^−1^ is probably associated with C–H bending modes and lipids [25,26].

Amide A (NH stretching) corresponds to the spectral region of 3300–3310 cm^−1^ [21,25]. The spectral band in the region of 1400 cm^−1^ is characteristic of the protein functional group attributed to the CH3 wagging mode [25]. Protein spectra are characterized by Amide stretching and bending vibrations.

Only minor changes in band intensity were observed after ozonation. The Amide A band slightly decreased, suggesting reduced bound water and hydrogen bonding. Conversely, the Amide I, II and III bands exhibited a slight increase in intensity, indicating subtle rearrangement of the collagen molecular environment while preserving the overall protein secondary structure. No significant peak shifts or new absorption bands were detected, suggesting that ozonation did not induce major biochemical degradation of the amniotic membrane.

**Table 1:** Functional group assignments and principal bands are detected in the HAM spectrum.

Functional Groups	ω (cm^−1^)	Assignments	References
Amide A	3292	ν NH	Sripriya, Kumar [21]; Talari et al. [25]
Lipids	2956	ν_a_ CH_3_	Fabian et al. [27]; Talari et al. [25].
Amide I	1639	ν C=O	Sripriya, Kumar [21]; Wang et al. [22]; Talari et al. [25]
Amide II	1548	sc N–H ν C–N	Sripriya, Kumar [21]; Socrates [23]
Lipids	1454	sc C–H	Talari et al. [25]; Santos et al. [26]
Proteins	1400	wag CH_3_	Talari et al. [25]
Amide III	1244	ν C–N sc N–H ν C–C sc C=O	Sripriya, Kumar [21]; Grdadolnik [24].

### 3.3. Histological Analysis of HAM

**Figure 4:**
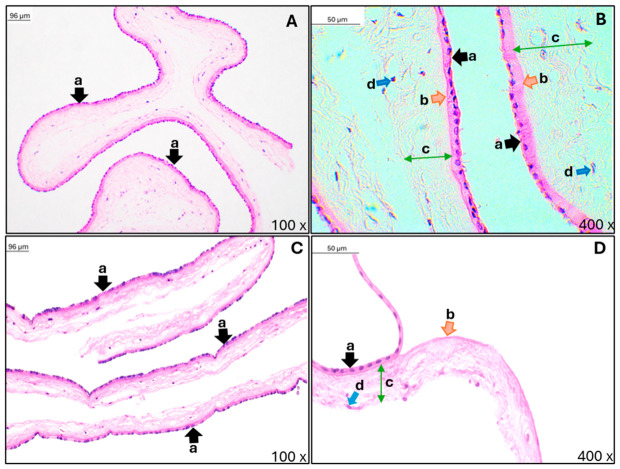
Representative histological images of human amniotic membrane stained with hematoxylin and eosin (H&E). Panels (**A**,**B**) correspond to the in natura membrane, whereas panels (**C**,**D**) correspond to the ozonated membrane. Black arrows (a) indicate epithelial cells, orange arrows (b) indicate the basement membrane, green arrows (c) indicate the stromal region, and blue arrows (d) indicate fibroblasts. Scale bars: 96 µm in panels (**A**,**C**), and 50 µm in panels (**B**,**D**).

The histological images of the samples are presented in Figure 4. Figure 4A,B show the images of the IN sample, in which a continuous epithelial layer with organized cuboidal cells adhered to one another can be observed, in addition to a well-defined basement membrane. The stromal structure appeared homogeneous, with organized collagen fibers and fibroblasts regularly distributed. These findings confirm the integrity of the membrane prior to treatment, indicating the preservation of its native structural composition.

Figure 4C,D present the images of the O_3_ sample, which was subjected to the hydrodynamic system with ozonated water for 40 min. The epithelial layer appeared more irregular when compared with the IN sample, with some regions exhibiting empty spaces and cells showing morphological modifications. The epithelial alterations did not compromise the extracellular matrix. The separation between epithelial cells and the widening of intercellular junctions indicates the occurrence of partial de-epithelization, a phenomenon that may be associated with the action of O_3_ on cellular interactions and tissue adhesion.

Despite these alterations, the basement membrane remained intact, demonstrating that the treatment did not compromise the fundamental structure of the HAM, which is an essential factor for its viability in biomedical applications.

### 3.4. Thermal Analyses by TGA and DSC

**Figure 5:**
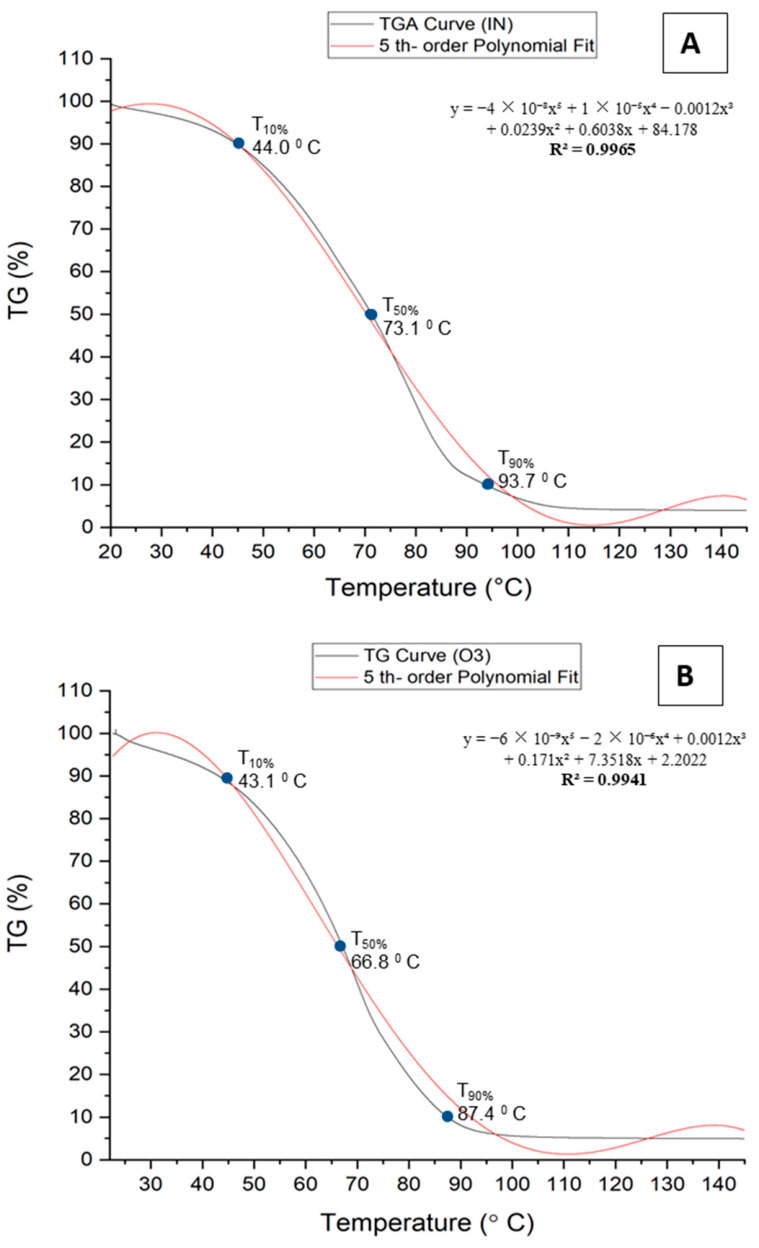
Thermogravimetric (TG) curves obtained for the IN-in natura (**A**) and O_3_-ozonated (**B**) samples. T10%, T50%, and T90% correspond to the temperatures at which 10%, 50%, and 90% mass loss occurred, respectively.

Figure 5 presents the comparative thermograms of the in natura (IN) and ozonated (O_3_) HAM samples. This analysis allows the evaluation of how sample mass varies as a function of temperature. The behavior of the samples was modeled using fifth-degree polynomial regression. The obtained R^2^ coefficient confirmed the accuracy of the fitting for both datasets.

In the comparative analysis of the temperatures required for 10% mass loss (T10%), this event occurred at 44.0 °C for the IN sample and at 43.1 °C for the O_3_ sample (∆ = −1.1 °C). Regarding the temperature corresponding to 50% mass loss (T50%), the IN sample exhibited this event at approximately 73.1 °C, whereas the O_3_ sample showed it at 66.8 °C (∆ = −6.3 °C). A similar profile was identified for 90% mass loss (T90%), which occurred at 93.7 °C in the IN sample and at 87.4 °C in the ozonated sample (∆ = −6.3 °C). Therefore, ozonation reduced the temperatures associated with 50% and 90% mass loss by 6.3 °C compared with the IN sample.

**Figure 6:**
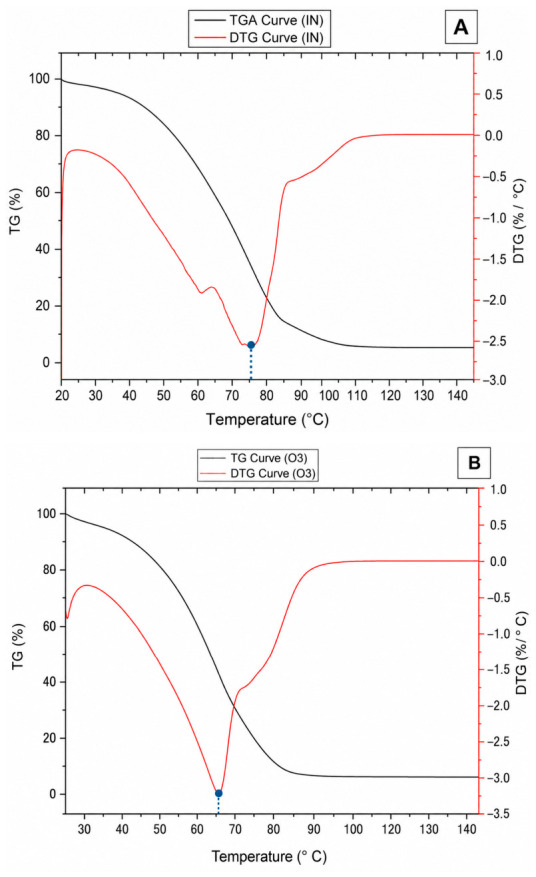
TG and DTG curves obtained for IN (**A**) and O_3_ (**B**) samples. The dashed lines in the graph highlight the temperature where the maximum mass loss rate (Tmax) occurred.

Figure 6 presents the TG-DTG curves of the hydrated IN (A) and O_3_ (B) samples. The data demonstrate that the IN sample (Figure 6A) exhibited a bimodal dehydration profile within the temperature range of 20 °C to 120 °C, indicating different energetic states of water within the biological matrix.

The first event, manifested as a thermal degradation shoulder around 60–65 °C, is attributed to the release of free and weakly bound water present on the biomaterial surface. The second event, more prominent and with a maximum mass loss rate centered at 80 °C, is correlated with the removal of primary hydration water, i.e., conjugated water. Thermal stabilization was observed at temperatures above 110 °C, at which point the thermogravimetric curve returned to the baseline plateau, indicating complete removal of free water and structurally bound water. At this stage, only the residual protein matrix (~5%) remained, corresponding to denatured collagen.

The DTG curve of the membrane subjected to ozone treatment (Figure 6B) exhibited a unimodal mass loss profile, characterized by a single prominent peak at approximately 70 °C. A shift in the peak temperature from 80 °C in the IN sample to 70 °C in the O_3_ sample was observed, corresponding to a reduction of 10 °C. After 100 °C, the mass loss rate returned to the baseline, indicating complete desiccation of the sample, with approximately 5% of the initial sample mass remaining.

**Figure 7:**
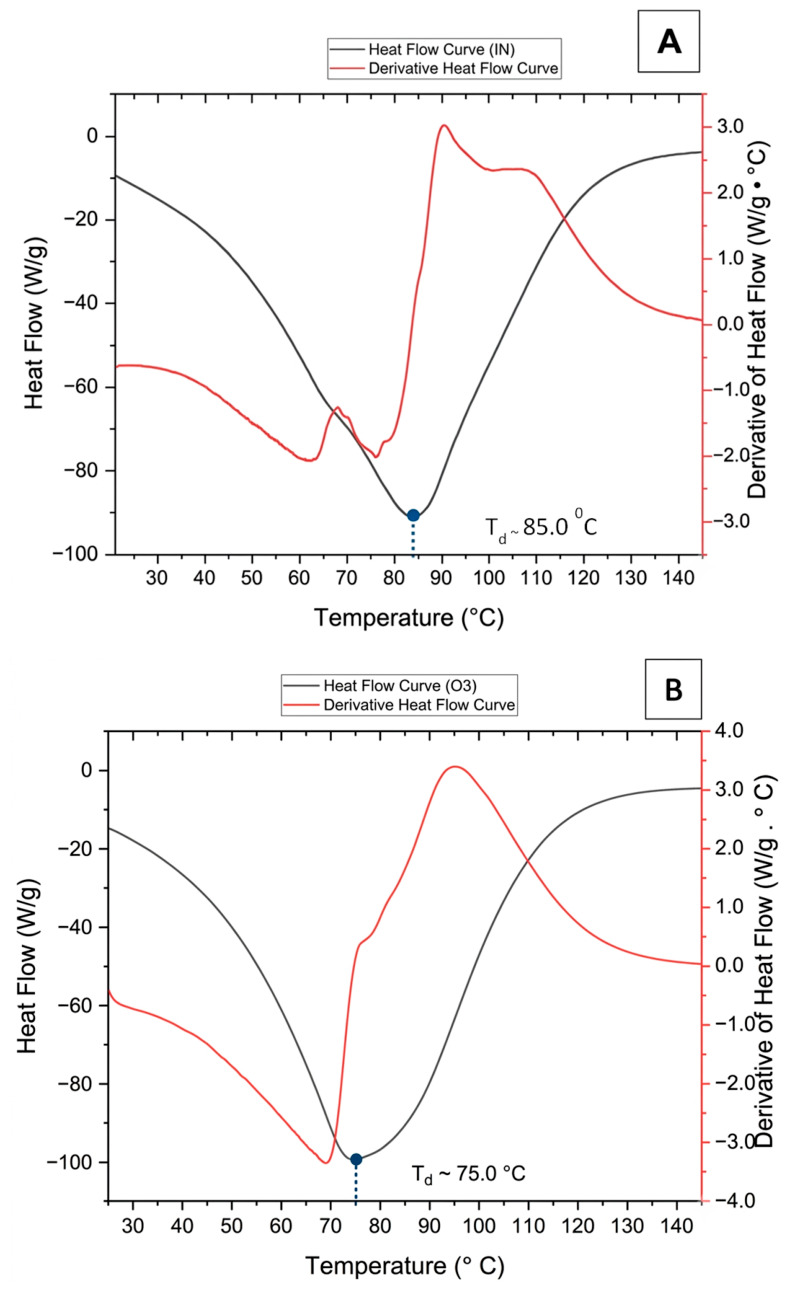
DSC-dDSC curves obtained for the hydrated IN (**A**) and O_3_ (**B**) samples. T_d_ represents the temperature of the endothermic denaturation peak.

The hydrated IN and O_3_ HAM samples were also characterized by DSC (Figure 7). The central event in the thermogram of the hydrated IN sample was a broad endothermic peak with a peak temperature at approximately 85 °C (Figure 7A). In contrast, the central event in the thermogram of the hydrated O_3_ sample was a broad endothermic peak with a peak temperature at approximately 75 °C (Figure 7B). In comparative analysis with the IN sample (Td~85 °C), a shift of the denaturation peak toward a lower denaturation temperature was observed in the O_3_ sample (Td~75 °C).

Analysis of the dDSC curve of the hydrated IN sample allowed the identification of energetic transitions associated with temperature variation. The derived thermogram exhibited an initial instability zone between 60 °C and 70 °C, characterized by oscillations in the energy absorption rate, attributed to the onset of disruption of the outermost and more volatile hydration layers. Furthermore, an abrupt transition regime beginning at approximately 75 °C was identified. This event reached its maximum magnitude of energetic variation between 80 °C and 90 °C. Above 110 °C, the curve returned to the baseline, indicating the completion of phase transition events and the complete conversion of the matrix into a dehydrated and structurally denatured state.

The dDSC analysis of the ozonated HAM sample (Figure 7B) exhibited a profile with a prominent unimodal event, with the point of maximum energetic variation shifted to approximately 70 °C.

The morphology of the dDSC curve confirms that the energetic barrier for denaturation was reduced, facilitating the conformational transition of the protein under lower thermal demands. After the peak around 70 °C, the derivative gradually returned to the baseline, stabilizing at approximately 110 °C, marking the end of the endothermic processes related to molecular reorganization and structural dehydration of the processed sample.

## 4. Discussion

The present study demonstrated that hydrodynamic ozonated water processing preserved the overall biochemical composition and structural integrity of the HAM, while inducing selective alterations in its epithelial layer and thermal behavior. FT-IR analysis confirmed the preservation of the characteristic collagen-related absorption bands, indicating that the molecular composition of the extracellular matrix remained unchanged after processing. Histological evaluation revealed partial epithelial disorganization and focal cell loss without compromising the basement membrane or stromal architecture. In addition, thermal analyses (TGA/DTG, DSC, and dDSC) demonstrated a transition from a bimodal to a unimodal dehydration profile, accompanied by a reduction in the temperatures required for water removal and collagen denaturation, indicating decreased thermal stability of the hydrated collagen matrix. Collectively, these findings suggest that hydrodynamic ozonated water processing primarily affects the hydration dynamics and epithelial compartment of HAM while maintaining the overall extracellular matrix organization, providing new insights into the physicochemical effects.

The FT-IR analysis performed in the present study demonstrated that hydrodynamic ozonated water processing did not induce significant changes in the biochemical profile of the HAM, as evidenced by the preservation of the characteristic absorption bands associated with collagen, lipids, carbohydrates, and nucleic acids. Although no major spectral changes were detected after hydrodynamic ozonated water processing, subtle variations in band intensity were observed. The Amide A band at approximately 3292 cm^−1^ showed a slight decrease in intensity, suggesting a reduction in bound water and/or changes in hydrogen bonding interactions. Conversely, the Amide I (1639 cm^−1^), Amide II (1548 cm^−1^), and Amide III (1244 cm^−1^) bands exhibited a slight increase in intensity, indicating subtle rearrangements in the collagen molecular environment while preserving the overall secondary structure of the extracellular matrix. Importantly, no significant peak shifts or new absorption bands were detected, indicating the absence of major biochemical degradation after processing. Similar results were reported by Santos et al. [26], who also observed preservation of the characteristic FT-IR bands after ozonation, indicating the absence of significant degradation of the collagen-rich extracellular matrix. However, in that study, HAM samples were exposed to ozonated water for 55 min, as the primary objective was to promote de-epithelialization rather than sterilization. Consequently, subtle spectral changes, including slight band shifts and intensity variations, were observed while the extracellular matrix remained structurally preserved. In contrast, the present study evaluated HAM processed for 40 min, which corresponds to the exposure time previously demonstrated to achieve effective sterilization using the same hydrodynamic ozonated water system [15]. Therefore, the present findings extend previous observations by demonstrating that the sterilization protocol preserves the biochemical composition of HAM, supporting the maintenance of its structural integrity after processing.

The histological analysis demonstrated that hydrodynamic ozonated water processing induced morphological alterations predominantly in the epithelial layer of the HAM, characterized by epithelial disorganization and partial loss of epithelial cells in some regions, while the basement membrane and stromal architecture remained preserved. These findings suggest that the treatment primarily affects the superficial cellular layer without causing extensive disruption of the underlying extracellular matrix. Similar observations have been reported in previous studies. Awoyama et al. [14] demonstrated that HAM exposed to ozone for 10 min preserved epithelial cell morphology, whereas a 15 min exposure resulted in more pronounced epithelial degeneration, although the basement membrane and stromal compartments remained structurally intact. Likewise, Kawata et al. [28] investigated the effects of a hydrodynamic ozonated water system applied for 15, 40, and 60 min and reported a significant reduction in epithelial thickness after 40 min of treatment, accompanied by epithelial disorganization and focal loss of the epithelial layer, as confirmed by histological and scanning electron microscopy analyses. The close agreement between these findings and the present results reinforces the hypothesis that hydrodynamic ozonated water processing preferentially affects the epithelial compartment while maintaining the structural integrity of HAM, an important characteristic for regenerative applications.

Thermogravimetric analysis demonstrated that the hydrated HAM exhibited an early mass loss event associated with water evaporation. The temperature corresponding to 10% mass loss was lower in the ozonated sample (87.5 °C) than in the in natura sample (93.7 °C), indicating that ozonated water processing reduced the thermal energy required to remove water from the biomaterial. This finding suggests modifications in water–matrix interactions, possibly resulting from subtle changes in the epithelial layer and collagen-associated hydrogen bonding, while preserving the overall structural integrity of the extracellular matrix. These results differ from those reported by Skopińska-Wisniewska et al. [29], who evaluated dehydrated HAM and described a two-stage thermal decomposition process, consisting of an initial dehydration event below 150 °C (5–20% mass loss) followed by protein degradation between 200 and 500 °C, with residual inorganic ash remaining above 600 °C. Because the present study investigated hydrated HAM, thermal analyses were intentionally limited to 150 °C to specifically characterize the dehydration process without inducing collagen degradation. To our knowledge, studies addressing the thermal behavior of hydrated HAM remain scarce, as most published investigations have focused on dehydrated or lyophilized membranes. Therefore, the present work provides novel information regarding the effects of hydrodynamic ozonated water processing on the hydration dynamics and low-temperature thermal behavior of hydrated HAM.

The thermal behavior observed in the present study can be explained by the structural organization of HAM and its hydration state. TGA and DSC analyses demonstrated that ozonated water processing reduced the temperature required for water removal and collagen denaturation, while preserving the overall structural organization of the extracellular matrix. These findings suggest that the treatment primarily affected water–matrix interactions rather than inducing extensive degradation of the collagen network. The stromal extracellular matrix of HAM is predominantly composed of collagen fibers, adhesive glycoproteins, and proteoglycans, which collectively maintain the three-dimensional architecture of the tissue and regulate cell–matrix interactions [30,31]. Among these components, type I and III collagens constitute the major fibrillar network responsible for the mechanical strength, elasticity, and structural integrity of the membrane, whereas type IV, V, and VI collagens are mainly localized in the basement membrane, contributing to epithelial attachment and stabilization of the epithelium–stroma interface [30,32]. De Simone et al. [17] demonstrated that water molecules associated with type I collagen play a fundamental role in stabilizing the triple collagen helix. According to these authors, free and bound water generate distinct hydration states that directly influence the thermal behavior of collagen, producing characteristic dehydration and endothermic events in hydrated samples that are absent after dehydration. This mechanism supports the present findings, in which alterations in dehydration and denaturation temperatures are likely associated with changes in collagen hydration rather than disruption of the extracellular matrix structure.

The TG-DTG thermograms obtained in the present study revealed distinct dehydration behaviors between the in natura (IN) and ozonated HAM. The hydrated IN sample exhibited a bimodal dehydration profile, indicating the presence of two energetically distinct water populations within the extracellular matrix. The first event, occurring between approximately 60 and 65 °C, was attributed to the evaporation of free or weakly bound water, whereas the second event, with a maximum mass loss rate at approximately 80 °C, corresponded to the removal of structural (bound) water associated with the collagen network. Above approximately 110 °C, the thermogravimetric curve reached a stable plateau, indicating that dehydration was essentially complete, leaving the dry organic matrix.

In contrast, the ozonated HAM exhibited a unimodal DTG profile, characterized by a single dehydration peak centered at approximately 70 °C. This shift toward lower temperatures indicates that less thermal energy was required to remove water from the biomaterial after processing, suggesting changes in the interactions between water molecules and the extracellular matrix. The merging of the two dehydration events into a single peak also indicates a reduction in the energetic distinction between free and bound water populations, reflecting decreased thermal stability of the hydrated collagen matrix.

These findings are consistent with the structural organization of HAM, in which the stromal extracellular matrix is predominantly composed of type I and III collagen fibers that retain water through extensive hydrogen bonding. De Simone et al. [17] demonstrated that free and bound water contribute differently to collagen stabilization, generating distinct dehydration events in thermal analyses of hydrated tissues. Therefore, the transition from a bimodal to a unimodal dehydration profile observed in the present study is likely associated with subtle alterations in water–collagen interactions induced by hydrodynamic ozonated water processing. Moreover, the partial disorganization of the epithelial layer observed histologically may have contributed to changes in water retention, facilitating water release at lower temperatures while preserving the overall integrity of the collagen-rich extracellular matrix.

Differential scanning calorimetry (DSC) demonstrated that hydrodynamic ozonated water processing altered the thermal behavior of HAM. The hydrated in natura (IN) sample exhibited a well-defined endothermic peak with a denaturation temperature (Td) of approximately 85 °C, whereas the ozonated sample showed a broader peak centered at approximately 75 °C. The 10 °C reduction in Td indicates that less thermal energy was required to induce collagen denaturation after treatment, suggesting a decrease in the thermal stability of the hydrated collagen matrix. Furthermore, the broader endothermic event observed in the ozonated sample is indicative of a less homogeneous thermal transition, consistent with subtle alterations in collagen–water interactions while preserving the overall extracellular matrix organization.

These findings are consistent with the known thermal behavior of collagen-rich biological tissues. DSC analysis characterizes the endothermic transition associated with the denaturation of the collagen triple helix, which is strongly influenced by the hydration state of the matrix [18]. In the IN sample, the onset of the endothermic transition occurred at approximately 60 °C, coinciding with the release of free water, whereas the maximum heat absorption at 85 °C corresponded to the cooperative disruption of the collagen triple helix following the removal of structurally bound water. The well-defined peak observed in the control group reflects the high thermal stability of native collagen and indicates preservation of its hydrated molecular organization before heating.

The lower denaturation temperature observed after ozonated water processing corroborates the TGA results, which also demonstrated a reduction in the temperature required for water removal. Together, these findings suggest that the treatment promoted subtle changes in water–collagen interactions, facilitating dehydration and collagen denaturation without evidence of extensive structural degradation. This interpretation is further supported by the FT-IR and histological analyses, which demonstrated preservation of the characteristic biochemical profile and overall stromal architecture despite partial alterations in the epithelial layer.

The derivative DSC (dDSC) analysis performed in the present study provided additional insight into the thermal transitions of HAM. The in natura (IN) sample exhibited two well-defined thermal events: an initial transition between approximately 60 and 70 °C, followed by a second, more pronounced event between 80 and 90 °C. The presence of these distinct transitions indicates that dehydration and collagen denaturation occurred through sequential thermal processes, reflecting the preserved structural organization of the hydrated extracellular matrix. The first event is associated with the progressive removal of free or weakly bound water, whereas the second corresponds to the disruption of interactions involving structurally bound water and the subsequent denaturation of the collagen network.

These findings are consistent with the DSC and TG-DTG results, which demonstrated a bimodal dehydration profile and a denaturation temperature of approximately 85 °C for the IN sample. Previous studies have shown that the thermal stability of collagen is strongly dependent on its hydration state, as structurally bound water stabilizes the collagen triple helix through hydrogen bonding [17,18]. Therefore, the clear separation of the two thermal events observed in the IN sample indicates that the energetic barrier associated with the removal of bound water remained preserved, allowing dehydration and collagen denaturation to occur as distinct but closely related processes.

The dDSC analysis performed in the present study demonstrated that hydrodynamic ozonated water processing modified the thermal transition profile of the HAM. Unlike the in natura (IN) sample, which exhibited two well-defined thermal events, the ozonated membrane presented a single broad transition centered at approximately 70 °C. This shift toward lower temperatures indicates that the thermal events associated with dehydration and collagen denaturation became less energetically distinct after treatment, suggesting a reduction in the thermal stability of the hydrated collagen matrix.

These findings are consistent with the TG-DTG and DSC analyses, both of which also demonstrated lower temperatures for water removal and collagen denaturation following ozonated water processing. The transition from a bimodal to a unimodal thermal profile suggests that hydrodynamic ozonated water processing altered the interactions between water molecules and the extracellular matrix, reducing the energetic barrier required for dehydration and subsequent collagen denaturation. This interpretation is further supported by the histological findings, which demonstrated partial disorganization of the epithelial layer while preserving the stromal architecture, indicating that the observed thermal changes are more likely associated with modifications in hydration dynamics.

One hypothesis that may explain the results obtained in the thermal analyses is that ozone processing induces alterations (disorganization/removal in some regions) in the HAM epithelial layer. Studies demonstrate that the HAM epithelial layer physiologically contributes to water retention and maintenance of amniotic microenvironment homeostasis, preserving the conditions necessary for fetal development [30,31,32]. The study by Dos Santos et al. [33] evaluated the percentage of water reduction in in natura HAM samples compared with samples subjected to ozonation using the same hydrodynamic system employed in the present work. The results demonstrated that ozonated samples exhibited a 34% greater water reduction than in natura samples, indicating that epithelial destabilization induced by ozonation favors the dehydration process. In this context, the disorganization/partial removal of the epithelium induced by ozonation may facilitate the loss of free and conjugated water, reducing the thermodynamic stability of the HAM.

The histological analysis using H&E staining provided important information regarding the general morphological preservation of HAM after hydrodynamic ozonated water treatment. However, this approach does not allow specific assessment of apoptotic pathways or detailed characterization of extracellular matrix proteins. Considering that ozone is a potent oxidizing agent capable of inducing reactive oxygen species and oxidative stress in biological tissues, additional investigations using apoptosis markers, such as cleaved CASPASE-3, would be valuable to determine whether the epithelial alterations observed after treatment are associated with apoptotic mechanisms. A recent study reported increased CASPASE-3 activation following ozone exposure, suggesting that oxidative stress-mediated apoptotic pathways may contribute to ozone-induced tissue responses [34]. Moreover, collagen-specific histochemical staining (e.g., Masson’s trichrome or Herovici’s stain) could provide further insights into the biological effects of the treatment on cellular integrity and collagen organization. Likewise, immunohistochemical analysis of key growth factors, including TGF-β, EGF, and VEGF, would be relevant to determine whether the regenerative bioactivity of HAM is preserved after processing [12]. Nevertheless, the maintenance of characteristic collagen-associated FT-IR bands and the preservation of thermal transition profiles observed by DSC and TGA suggest that the matrix architecture remained stable after treatment. These findings support the hypothesis that the ozonated water protocol may preserve essential matrix properties, although further molecular and functional investigations are still required.

From a clinical application perspective, it should be considered that several studies in the literature recommend HAM de-epithelization to optimize its function as a scaffold for ocular surface reconstruction, which is indicated for the treatment of different ocular diseases [35,36]. De-epithelization of the human amniotic membrane exposes its basement membrane, creating a highly favorable microenvironment for cell adhesion and proliferation. This characteristic confers relevance to the tissue not only in ophthalmological applications but also in different areas of regenerative medicine [37,38].

## 5. Conclusions

This study provides the first comprehensive characterization of the effects of hydrodynamic ozonated water processing on hydrated HAM by integrating morphological, biochemical, and thermal analyses. The results demonstrate that this processing protocol preserves the biochemical composition and collagen-rich extracellular matrix while inducing controlled epithelial alterations and moderate reductions in thermal stability associated with changes in water–collagen interactions. These findings indicate that the proposed protocol maintains the structural features required for the use of HAM as a biological scaffold while providing new insights into the physicochemical effects of ozonated water processing. Nevertheless, further investigations addressing mechanical performance, preservation of bioactive molecules, and in vitro and in vivo biological responses are necessary to establish its clinical applicability in regenerative medicine.

## Data Availability

The original contributions presented in the study are included in the article, further inquiries can be directed to the corresponding author.
